# Study on Microstructure and Mechanical Properties of Hypereutectic Al–18Si Alloy Modified with Al–3B

**DOI:** 10.3390/ma11030456

**Published:** 2018-03-20

**Authors:** Chunjie Gong, Hao Tu, Changjun Wu, Jianhua Wang, Xuping Su

**Affiliations:** 1 Jiangsu Key Laboratory of Materials Surface Science and Technology, Changzhou University, Changzhou 213164, China; gongchunjie2000@foxmail.com (C.G.); tuhao@cczu.edu.cn (H.T.); wucj@cczu.edu.cn (C.W.); sxping@cczu.edu.cn (X.S.); 2 Jiangsu Collaborative Innovation Center of Photovoltaic Science and Engineering, Changzhou University, Changzhou 213164, China; 3 National Experimental Teaching Demonstration Center of Material Science and Engineering, Changzhou University, Changzhou 213164, China

**Keywords:** hypereutectic Al–18Si alloy, modification, microstructure, mechanical properties

## Abstract

An hypereutectic Al–18Si alloy was modified via an Al–3B master alloy. The effect of the added Al–3B and the modification temperature on the microstructure, tensile fracture morphologies, and mechanical properties of the alloy were investigated using an optical microscope, Image–Pro Plus 6.0, a scanning electron microscope, and a universal testing machine. The results show that the size of the primary Si and its fraction decreased at first, and then increased as an additional amount of Al–3B was added. When the added Al–3B reached 0.2 wt %, the fraction of the primary Si in the Al–18Si alloy decreased with an increase in temperature. Compared with the unmodified Al–18Si alloy, the tensile strength and elongation of the alloy modified at 850 °C with 0.2 wt % Al–3B increased by 25% and 81%, respectively. The tensile fracture of the modified Al–18Si alloy exhibited partial ductile fracture characteristics, but there were more areas with ductile characteristics compared with that of the unmodified Al–18Si alloy.

## 1. Introduction

Hypereutectic Al–Si alloys have excellent properties such as low thermal expansion, high wear resistance, high corrosion resistance, small specific gravity, and good thermal conductivity [1,2]. These alloys are an excellent engine piston material, which is widely used in aerospace, shipbuilding, and the automobile industry, in addition to other industries [3]. The primary Si in an hypereutectic Al–Si alloy is usually either shaped like a five–petal star, a plate, or an octahedron. The eutectic Si of the alloy is present as a thick needle [4]. The wear resistance of the alloy can be improved by the existence of the primary Si. However, the angle–shaped primary Si drastically cuts the matrix, which reduces the mechanical properties of the alloy [5,6]. Therefore, it is of great importance to improve the mechanical properties of an hypereutectic Al–Si alloy by refining the primary Si, which could expand its application field.

Usually, methods to refine primary Si in hypereutectic Al–Si alloys include ultrasonic vibration [7], pressure casting [8], rotating magnetic field stirring [9], high and low–temperature melting mixtures [10], and rapid solidification [11] and modification [4]. Modifications have been widely used, because they are simple to operate and inexpensive to perform. The alloy is usually modified with phosphorus [12], sodium [13], and rare earth elements [14]. Most studies have focused on how modifications affect the size and morphology of the primary Si. The effects of modification on the non–equilibrium eutectic point movements of the Al–Si alloy and the resulting microstructure and mechanical property changes of the alloy have rarely been studied. Our previous studies demonstrated that the hypereutectic structures in hypoeutectic ZZnAl4Y alloy could be obtained through Al–5Ti–B modification [15]. The mechanical properties of the alloy were significantly improved after modification. Jiang et al. [16] quantitatively estimated the shifting distance of the eutectic point in hypereutectic Al–Si alloys using mathematical calculations.

In the present work, an Al–18Si alloy was modified with an Al–3B master alloy. The effects of the modification on the movement of the Al–Si alloy non–equilibrium eutectic points and the resulting microstructure and mechanical property changes of the Al–18Si alloy were investigated. The importance of this research lies in the expansion of alloy applications for the alloy industry.

## 2. Experimental Section

The raw materials used in this experiment were industrial pure aluminum (A00, purity of 99.85%), Al–50Si, and Al–3B master alloy. First, the pure aluminum and Al–50Si master alloy were mixed, and then melted at 850 °C to obtain 4000 g ingots of Al–18Si alloy. In order to investigate the effect of temperature and added amounts of Al–3B on modification of the Al–18Si alloy, 200 g of the Al–18Si alloy was remelted at different temperatures for 30 min, and then modified based on the process parameters, as shown in Table 1. During the melting process, a coating agent of NaCl:KCl:Na_3_AlF_6_ = 6:9:5 was used to protect the liquid alloy from oxidation. An Al–3B master alloy wrapped in aluminum foil was pressed into the alloy liquid for 5 min, and then constantly stirred for 30 s. After modification, 0.1 wt % C_2_Cl_6_ was used to remove gas and slag from the melt. The modified Al–18Si alloy liquid was poured into a 100 °C mild steel mold with an inner size of Φ12 mm × 100 mm.

The samples for metallographic analysis were cut at a position of 10 mm from the bottom of the casting samples. These samples were polished to a mirror finish using Al_2_O_3_, and further etched with Keller’s reagent. The microstructural characterization was obtained via a Leica DIM 3000 optical microscope (OM) (Solms, Germany). The area fraction of primary Si was calculated by the software Image–Pro Plus 6.0 (Media Cybernetics, Rockville, MD, USA). The mechanical properties of the Al–18Si alloy were measured by a WDW–300 universal tensile testing machine (kaiqiangli, Shenzhen, China) following the ASTM E8 Standard [17], and the strain rate was 10^−3^ S^−1^. Three samples were conducted per alloy, and the average value was determined. The characterization of the fracture morphologies of the tensile samples of the Al–18Si alloy was carried out with a JSM–6360LV scanning electron microscope (SEM) (JEOL, Tokyo, Japan).

## 3. Results and Discussion

### 3.1. Effect of Added Amount of Al–3B on Al–18Si Alloy Microstructure

**Figure 1:**
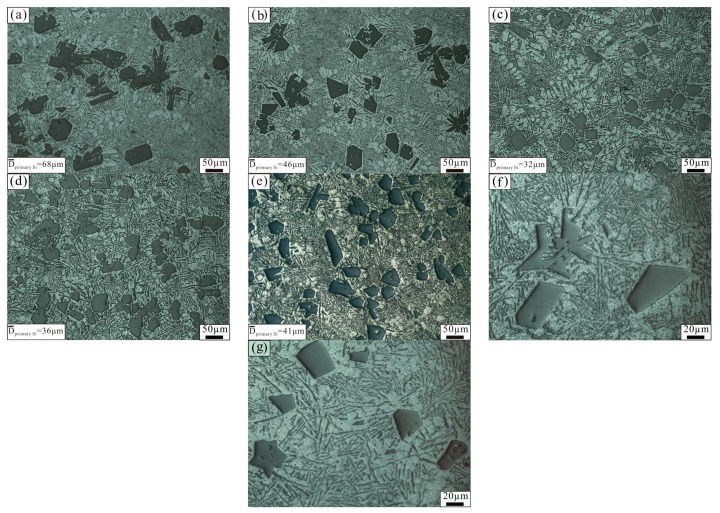
Microstructure of an Al–18Si alloy modified with different amounts of Al–3B: (**a**) 0 wt %; (**b**) 0.1 wt %; (**c**) 0.2 wt %; (**d**) 0.4 wt %; and (**e**) 0.6 wt %; (**f**) Higher magnification of (**a**); (**g**) Higher magnification of (**c**).

**Figure 2:**
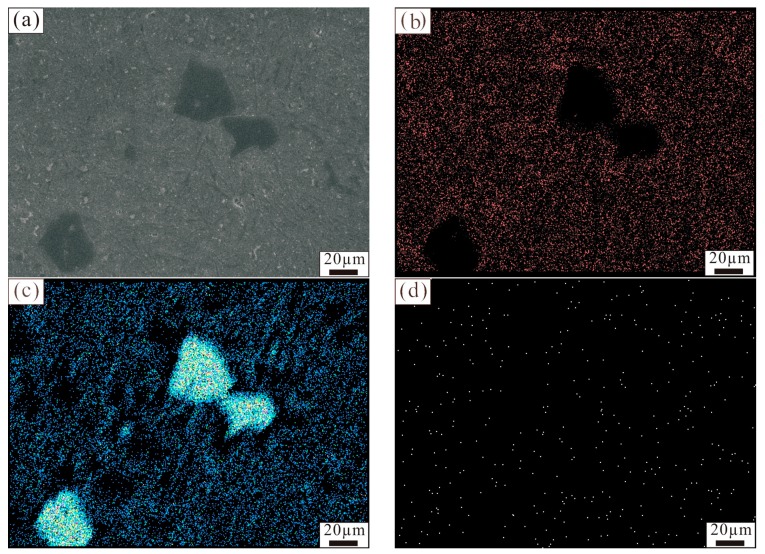
The area maps of the elements distribution in the modified Al–18Si alloy. (**a**) Microstructure of the Al–18Si alloy; (**b**) Map of Al distribution; (**c**) Map of Si distribution; (**d**) Map of B distribution.

Figure 1 shows the microstructure of the Al–18Si alloy modified at 850 °C with different amounts of Al–3B. Primary Si existed in the form of a large block in the unmodified Al–18Si alloy, as shown in Figure 1a. The average size of the primary Si was 68 μm, and the biggest one was more than 100 μm. When the added amount of Al–3B was 0.1 wt % and 0.2 wt %, the average size of the primary Si decreased to 46 μm and 32 μm, respectively. After that, the average size of the primary Si increased slightly, as the additional amount of Al–3B was added. When the added amount of Al–3B increased up to 0.4 wt % and 0.6 wt %, the average size of the primary Si was 36 μm and 41 μm, respectively. In addition, the number of α–Al in the Al–18Si alloy increased with each addition of Al–3B, as shown in Figure 1. When Al–3B was more than 0.2 wt %, the number of α–Al in the alloy no longer increased. The area maps of element (Al, Si, and B) distribution in the Al–18Si alloy modified at 850 °C with 0.2 wt % Al–3B are illustrated in Figure 2. It could be seen clearly from Figure 2 that the primary Si, α–Al, and eutectic Si exist in the Al–18Si alloy. Element B was evenly distributed in the modified alloy.

Table 2 shows the fraction of primary Si in the Al–18Si alloy before and after modification at different temperatures and with different amounts of Al–3B. From Table 2, it can be seen that the fraction of the primary Si in Al–18Si alloy first decreased, and then increased as the additional amount of Al–3B was added at 750 °C, 800 °C, and 850 °C, respectively. When the amount of Al–3B was 0.2 wt %, the fraction of the primary Si was the least.

Two mechanisms explain the variation in the fraction of the primary Si in the Al–18Si alloy as the Al–3B master alloy was added.

The first explanation is as follows. After Al–3B was added to the melted Al–18Si alloy, AlB_2_ particles in the Al–3B alloy might have promoted the nucleation and growth of the α–Al phase [18,19]. In turn, the nucleation and growth of the primary Si could be suppressed, resulting in a decrease in the primary Si fraction. When the added amount of Al–3B was more than 0.2 wt %, the modification effect would no longer be enhanced. However, because of the decrease in the melting temperature, the cooling rate of the melted alloy also decreased. As a result, the primary Si in the melted alloy would have time to grow, and the fraction of the primary Si in the resultant alloy would finally be increased. 

**Figure 3:**
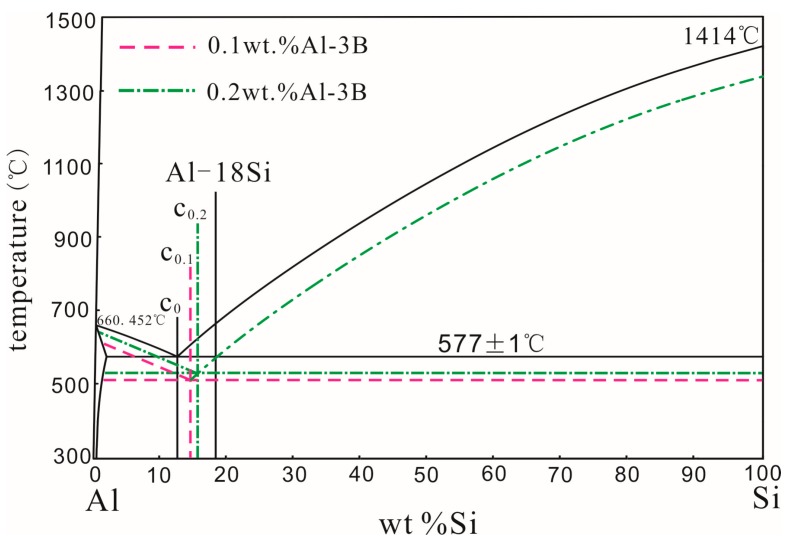
Schematic of eutectic point variation of an Al–Si alloy modified with a smaller amount of Al–3B.

The second explanation is that the non–equilibrated eutectic point of the Al–Si alloy should be moved after modification with Al–3B. In the Al–Si binary equilibrium phase diagram, the eutectic point is the intersection of two liquidus. If the liquid alloy solidifies at a faster rate, the liquidus of hypoeutectic and hypereutectic alloys should move downward at the same extent. At this time, the position of the transverse coordinates of the non–equilibrium eutectic point is the same as that of the equilibrium eutectic point. If the liquid alloy is modified with Al–3B, the liquidus of the hypoeutectic alloy should move upward to some extent, but the liquidus of the hypereutectic alloy should hardly move. As a result, the position of the transverse coordinates of the non–equilibrium eutectic point should move to the right. Figure 3 shows a schematic diagram of the eutectic point variation in the Al–Si alloy modified at 850 °C with a smaller amount of Al–3B. In Figure 3, C_0_ represents the equilibrated eutectic point in the Al–Si binary phase diagram. C_0.1_ and C_0.2_ represent the eutectic points at non–equilibrium states that correspond to the melted alloys modified with 0.1 wt % and 0.2 wt % Al–3B, respectively. When the amount of added Al–3B was smaller, modifications had little effect on the melting temperature. The variation of the melting temperature together with its degree of undercooling, caused by the addition of Al–3B, could be ignored. Therefore, the undercooling degree of the hypereutectic Al–Si alloy can be considered constant. The undercooling degree of the hypoeutectic Al–Si alloy was smaller than that of the hypereutectic Al–Si alloy, due to modifications of Al–3B. As Al–3B additions increased, the number of nucleation cores of the α–Al phase also increased, resulting in a decrease in the undercooling degree of the hypoeutectic Al–Si alloy, as shown in Figure 3. With this decrease, the non–equilibrated eutectic point would move to the right. According to the Lever’s law, the fraction of primary Si should decrease as more Al–3B is added.

**Figure 4:**
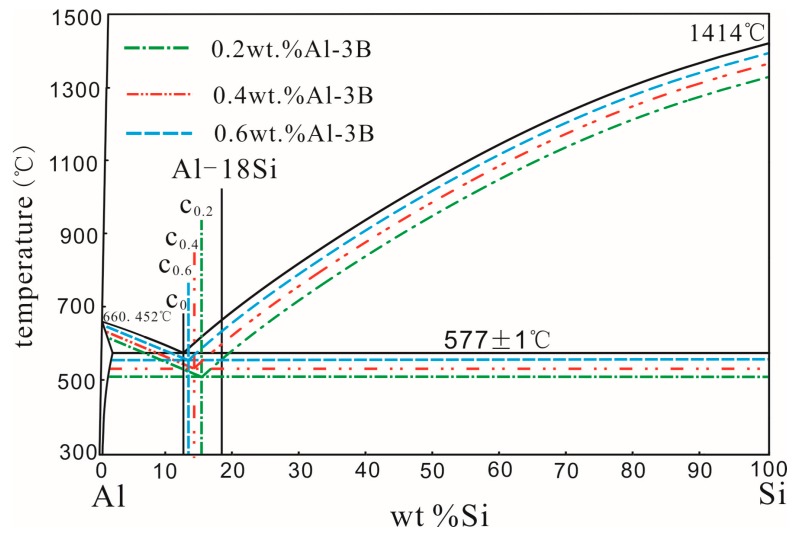
Schematic of eutectic point variation of an Al–Si alloy modified with a higher amount of Al–3B.

Figure 4 is a schematic diagram of the Al–Si alloy eutectic point variations modified at 850 °C with higher amounts of Al–3B. In Figure 4, C_0.2_, C_0.4_, and C_0.6_ represent the non–equilibrated eutectic points, corresponding to the melted alloy modified with 0.2 wt %, 0.4 wt %, and 0.6 wt % Al–3B, respectively. When the amount of Al–3B is greater, the addition of a modifier on the temperature of the melted alloy and the degree of undercooling should be considered. With increased Al–3B additions, the temperature of the melted alloy and its cooling rate decreased, which resulted in a decreased undercooling degree, as shown in Figure 4. Moreover, due to the Al–3B modification effects, the undercooling degree of the hypoeutectic Al–Si alloy was much smaller than that of the hypereutectic Al–Si alloy. Therefore, as the added amount of Al–3B increased, the non–equilibrated eutectic point of the Al–Si alloy moved continuously to the left. According to the Lever’s law, the fraction of the primary Si should increase with the addition of more Al–3B.

### 3.2. The Effect of Temperature on an Hypereutectic Al–18Si Alloy Microstructure

**Figure 5:**
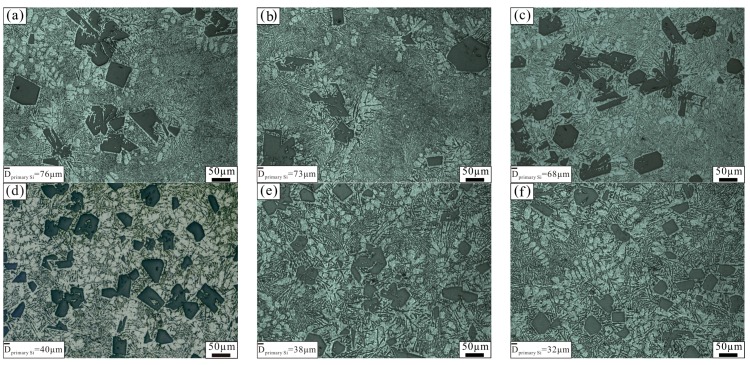
Microstructure of the unmodified Al–18Si alloy and the alloy modified at different temperatures: (**a**) 750 °C, unmodified; (**b**) 800 °C, unmodified; (**c**) 850 °C, unmodified; (**d**) 750 °C, modified; (**e**) 800 °C, modified; (**f**) 850 °C, modified.

Figure 5 shows the microstructure of the unmodified Al–18Si alloy and the alloy modified with 0.2 wt % Al–3B at different temperatures. As can be seen in Figure 5, the number of α–Al in the Al–18Si alloy increased, and the size of primary Si decreased obviously after modification. As the melting temperature increased, the size of the primary Si in the unmodified alloy barely changed; however, marked changes were seen in the size of the primary Si in the modified alloy. When the modification temperature increased from 750 °C to 800 °C, the size of the primary Si in the modified alloy barely changed. After the modification temperature increased to 850 °C, the primary Si in the modified alloy was obviously refined. The average size of the primary Si in the unmodified and modified Al–18Si alloys was 68 μm and 32 μm, respectively. Studies have confirmed that the melted hypereutectic Al–Si alloy is heterogeneous [20,21,22]: many Si–rich regions and small primary Si particles were found in the melted alloy at lower melting temperatures. When the Al–18Si alloy was melted and modified at 750–800 °C, numerous tiny primary Si particles and Si–rich regions were found in the melted alloy. The effect of Al–3B modification was poor because of the lower Si content in the liquid alloy. When the modification temperature was 850 °C, the initial primary Si particles and Si–rich regions in the melted alloy were completely dissolved, because the Al–18Si alloy was sufficiently overheated. As a result, the Si content of the liquid alloy was basically equal to the total Si content of the alloy. Therefore, the effect of Al–3B modification on the Al–18Si alloy can be enhanced. After solidification of the Al–18Si alloy, numerous fine primary Si particles were formed.

As shown in Table 2, if the added amount of Al–3B is fixed, the fraction of primary Si in Al–18Si decreases as the modification temperature increases.

**Figure 6:**
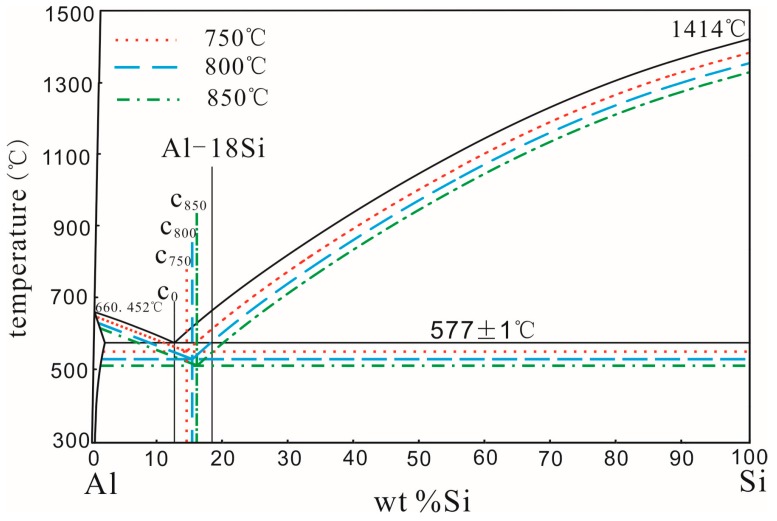
Schematic of eutectic point variation of the Al–Si alloy modified by 0.2 wt % Al–3B at different temperatures.

How the temperature affects the fraction of the primary Si in the modified Al–18Si alloy can be explained by the movement of the Al–Si alloy’s non–equilibrated eutectic point. According to the mechanisms of Al–5Ti master alloy modification on Al–12.6Si alloy that Wang et al. proposed [23], a schematic of the eutectic point variation of an Al–Si alloy modified by 0.2 wt % Al–3B at different temperatures is given in Figure 6. In the figure, C_0_ is the equilibrium eutectic point of the Al–Si alloy, while C_750_, C_800_, and C_850_ represent non–equilibrated eutectic points that correspond to the melted alloy modified at 750 °C, 800 °C, and 850 °C, respectively.

When the Al–18Si alloy was modified with the Al–3B master alloy, the formation of the primary α–Al phase was enhanced. After Al–3B modification, the undercooling degree of the hypoeutectic Al–Si alloy should be decreased. Besides, with increased modification temperatures, the difference between the melted Al–18Si and metal mold temperatures also increased, which results in increased cooling rates and undercooling degrees, as shown in Figure 6. Due to the comprehensive effect of the Al–3B modifications and the variations in melting temperatures, the undercooling degree of the hypoeutectic Al–Si alloy was smaller than that of the hypereutectic Al–Si alloy. As a result, the Al–Si alloy non–equilibrated eutectic point moved to the right. Furthermore, the distance that the non–equilibrated eutectic point moves should increase with increased modification temperatures. According to the Lever’s law, the fraction of primary Si in the modified Al–18Si alloy decreased as the modification temperature increased.

### 3.3. The Mechanical Properties of an Hypereutectic Al–18%Si Alloy

Table 3 shows the mechanical properties of the unmodified Al–18Si alloy and the alloy modified with different amount of Al–3B at 750 °C, 800 °C, and 850 °C, respectively. With the increase of added amount of Al–3B, the tensile strength and elongation of Al–18Si alloy increased firstly, and then decreased at all of the modification temperatures. The mechanical properties of the alloy modified with 0.2 wt % Al–3B were the best. The tensile strength and elongation of the Al–18Si alloy modified at 850 °C were higher than that at 750 °C and 800 °C. The tensile strength and elongation of the modified alloy increased by 25% and 81% compared with the unmodified Al–18Si alloy, respectively.

**Figure 7:**
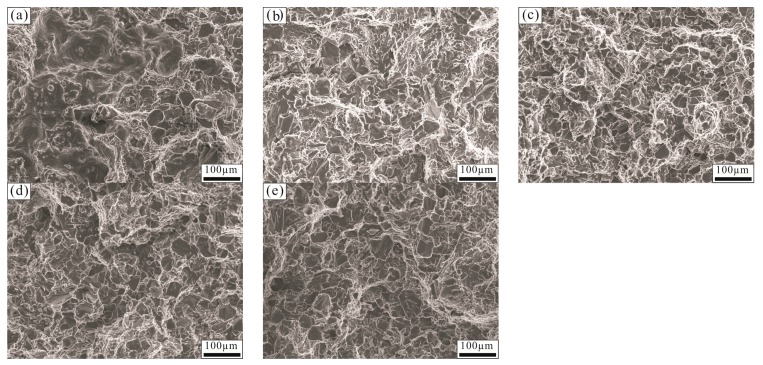
Fracture morphologies of the tensile samples of the Al–18Si alloy (**a**) melted at 850 °C and unmodified; (**b**) modified at 850 °C with 0.1 wt % Al–3B; (**c**) modified at 850 °C with 0.2 wt % Al–3B; (**d**) modified at 850 °C with 0.4 wt % Al–3B; and (**e**) modified at 850 °C with 0.6 wt % Al–3B.

SEM images of the tensile fracture surfaces of the Al–18Si alloy are shown in Figure 7. The tensile fracture of the modified Al–18Si alloy exhibited partial ductile fracture characteristics, but there were more areas of ductile characteristics compared with that of the unmodified Al–18Si alloy. As shown in Figure 7a, large cleavage steps, a few dimples, and torn edges were present on the tensile fracture surface of the unmodified Al–18Si alloy, which showed partial ductile fracture characteristics. After being modified at 850 °C with 0.1 wt % Al–3B, more dimples and torn edges were seen on the tensile fracture surface of the Al–18Si alloy, as shown in Figure 7b. The fracture surface of the Al–18Si alloy modified with 0.2 wt % Al–3B showed many ductile fracture characteristics; it had the highest number of the dimples and torn edges, as shown in Figure 7c, which is why the tensile strength and elongation of the modified Al–18Si alloy was greater than that of the unmodified alloy. After being modified at 850 °C with 0.4 wt % and 0.6 wt % Al–3B, respectively, the dimples and torn edges of the Al–18Si alloy decreased, as shown in Figure 7d,e, which also showed partial ductile fracture characteristics.

## 4. Conclusions

1. The size and area fraction of the primary Si in the Al–18Si alloy first decreased, and then increased as Al–3B was continuously added. When Al–18Si alloy was modified with 0.2 wt % Al–3B, the size and area fraction of the primary Si was the least.
2. The size of the primary Si in the modified Al–18Si alloy hardly changed as the temperature increased from 750 °C to 800 °C. As the temperature increased to 850 °C, the primary Si was obviously refined. As the modification temperature increased, the fraction of primary Si in the modified Al–18Si alloy gradually decreased.
3. After modification at 850 °C with 0.2 wt % Al–3B, the tensile strength and elongation of the modified Al–18Si alloy increased by 25% and 81%, respectively. The tensile fracture of modified Al–18Si alloy exhibited partial ductile fracture characteristics, but there were more areas of ductile characteristics compared with that of the unmodified Al–18Si alloy.

## Figures and Tables

**Table 1:** Modification temperature of the Al–18Si alloy and the added amount of Al–3B.

Alloy No.	Modification Temperature/°C	Adding Amount of Al–3B/wt %
1	750	0
2	750	0.1
3	750	0.2
4	750	0.4
5	750	0.6
6	800	0
7	800	0.1
8	800	0.2
9	800	0.4
10	800	0.6
11	850	0
12	850	0.1
13	850	0.2
14	850	0.4
15	850	0.6

**Table 2:** Area fraction of primary Si in the Al–18Si modified at different temperatures with different amounts of Al–3B (%).

Modification Temperature/°C	Added Amount of Al–3B (wt %)				
	0	0.1	0.2	0.4	0.6
750	14.5	12.8	11.5	13.2	14.7
800	13.6	11.5	9.4	12.3	13.9
850	12.3	10.0	8.6	11.1	12.8

**Table 3:** Mechanical properties of Al–18Si before and after modification.

Modification Temperature (°C)	Tensile Strength (SD) (N/mm^2^)					Elongation (SD) (%)				
	Added Amount of Al–3B (wt %)									
	0	0.1	0.2	0.4	0.6	0	0.1	0.2	0.4	0.6
750	141 (±2.9)	145 (±3.1)	166 (±2.9)	148 (±2.8)	142 (±2.5)	1.6 (±0.12)	2.2 (±0.13)	2.7 (±0.10)	2.3 (±0.14)	2.0 (±0.12)
800	143 (±2.8)	148 (±2.4)	164 (±2.7)	149 (±3.1)	144 (±2.6)	2.0 (±0.11)	2.3 (±0.13)	2.6 (±0.14)	2.4 (±0.10)	2.2 (±0.13)
850	142 (±3.0)	153 (±3.3)	178 (±2.8)	156 (±2.6)	147 (±3.6)	2.1 (±0.12)	2.7 (±0.14)	3.8 (±0.13)	2.9 (±0.12)	2.5 (±0.10)
